# Outdoor PM_2.5_, Ambient Air Temperature, and Asthma Symptoms in the Past 14 Days among Adults with Active Asthma

**DOI:** 10.1289/EHP92

**Published:** 2016-07-06

**Authors:** Maria C. Mirabelli, Ambarish Vaidyanathan, W. Dana Flanders, Xiaoting Qin, Paul Garbe

**Affiliations:** 1 Air Pollution and Respiratory Health Branch, and; 2 Environmental Health Tracking Branch, National Center for Environmental Health, Centers for Disease Control and Prevention, Atlanta, Georgia, USA; 3 Department of Epidemiology, Rollins School of Public Health, Emory University, Atlanta, Georgia, USA

## Abstract

**Background:**

Relationships between air quality and health are well-described, but little information is available about the joint associations between particulate air pollution, ambient temperature, and respiratory morbidity.

**Objectives:**

We evaluated associations between concentrations of particulate matter ≤ 2.5 μm in diameter (PM2.5) and exacerbation of existing asthma and modification of the associations by ambient air temperature.

**Methods:**

Data from 50,356 adult respondents to the Asthma Call-back Survey from 2006–2010 were linked by interview date and county of residence to estimates of daily averages of PM2.5 and maximum air temperature. Associations between 14-day average PM2.5 and the presence of any asthma symptoms during the 14 days leading up to and including the interview date were evaluated using binomial regression. We explored variation by air temperature using similar models, stratified into quintiles of the 14-day average maximum temperature.

**Results:**

Among adults with active asthma, 57.1% reported asthma symptoms within the past 14 days, and 14-day average PM2.5 ≥ 7.07 μg/m3 was associated with an estimated 4–5% higher asthma symptom prevalence. In the range of 4.00–7.06 μg/m3 of PM2.5, each 1-μg/m3 increase was associated with a 3.4% [95% confidence interval (CI): 1.1, 5.7] increase in symptom prevalence; across categories of temperature from 1.1 to 80.5°F, each 1-μg/m3 increase was associated with increased symptom prevalence (1.1–44.4°F: 7.9%; 44.5–58.6°F: 6.9%; 58.7–70.1°F: 2.9%; 70.2–80.5°F: 7.3%).

**Conclusions:**

These results suggest that each unit increase in PM2.5 may be associated with an increase in the prevalence of asthma symptoms, even at levels as low as 4.00–7.06 μg/m3.

**Citation:**

Mirabelli MC, Vaidyanathan A, Flanders WD, Qin X, Garbe P. 2016. Outdoor PM2.5, ambient air temperature, and asthma symptoms in the past 14 days among adults with active asthma. Environ Health Perspect 124:1882–1890; http://dx.doi.org/10.1289/EHP92

## Introduction

Ambient particulate matter (PM) pollution accounted for an estimated 3.1 million deaths and 3% of global disability-adjusted life years in 2010 (Lim et al. 2012). Health studies examining the effects of PM exposures have identified links between PM and new-onset asthma (Young et al. 2014), respiratory symptoms (Balmes et al. 2014; Young et al. 2014), hospitalizations and emergency department visits (Bell et al. 2008; Belleudi et al. 2010; Dominici et al. 2006), and death (Dominici et al. 2000; Samet et al. 2000; Zanobetti and Schwartz 2009) among adults. The biologic effects of particulate air pollution on respiratory health are determined largely by the size and composition of the particulate air pollution, deposition of the particles in the respiratory tract, and the immunologic response to the particles (Koren 1995). Mechanisms by which exposure to particulate air pollution may exacerbate respiratory health among individuals with asthma include oxidative stress, airway inflammation, and hyperresponsiveness of the airways (Bernstein et al. 2004; Li et al. 2003; Nel et al. 2001). In recognition of the importance to public health of the effects of particulate air pollution exposures, standards such as the California Air Resources Board Air Quality Standards, European Union Directives, U.S. National Ambient Air Quality Standards, and World Health Organization Air Quality Guidelines have been used throughout the world to establish ambient air quality standards (Vahlsing and Smith 2012).

A growing body of epidemiologic literature also provides initial evidence that exacerbations of adult asthma may be associated with ambient meteorological conditions, and in particular with temperature extremes (Beard et al. 2012; Fitzgerald et al. 2014; Michelozzi et al. 2009). However, the mechanisms through which outdoor temperature exposures may plausibly affect the respiratory tract remain unclear. Potential mechanisms include effects of temperature or other ambient conditions on dehydration and hyperosmolarity of the airways, which may directly induce exacerbations of asthma by triggering bronchoconstriction (Anderson and Daviskas 2000). Proposed indirect effects focus on exposures associated with temperature; for example, well-described associations between ozone (O_3_) and airway inflammation, particularly among adults with asthma (Hernandez et al. 2010; Khatri et al. 2009; Koren 1995), raise the possibility that the observed associations between temperature and exacerbations of asthma may be attributed, at least in part, to changes in the production of ground-level O_3_ that are correlated with temperature and other meteorological factors (Baur et al. 2004; Camalier et al. 2007). Higher temperatures are also associated with increased air pollutant emissions; for example, hot spells can lead to escalated use of air conditioning, thereby increasing demand on electricity-generating units, which can in turn lead to increased emissions of oxides of nitrogen (NO_x_) on hot days (He et al. 2013). The proposed pathways between temperature and exacerbations of asthma may also be modified by adaptive behaviors such as air conditioning use or avoidance of outdoor activities (Andrade et al. 2011). In the absence of clearly delineated mechanisms by which temperature is associated with mortality across the entire range of ambient outdoor temperatures, numerous recent studies have been designed to evaluate interactions between particulate air pollution and temperature that affect mortality, including respiratory, cardiovascular, and all-cause mortality (Breitner et al. 2014; Li et al. 2011; Nawrot et al. 2007; Park et al. 2011).

To date, however, little information is available about the joint associations between particulate air pollution, ambient temperature, and respiratory morbidity. Data from the Centers for Disease Control and Prevention’s (CDC’s) Environmental Public Health Tracking Network and the Behavioral Risk Factor Surveillance System (BRFSS) adult Asthma Call-back Survey provide a unique opportunity to further explore such a possibility by evaluating associations between ambient PM and asthma symptoms among adults with asthma in a large and geographically diverse sample of adults. In the present study, we combined modeled county-level estimates of ambient concentrations of PM ≤ 2.5 μm in aerodynamic diameter (PM_2.5_), ambient concentrations of O_3_, precipitation, and air temperature with individual-level characteristics of adults with asthma to evaluate associations between PM_2.5_ and asthma exacerbations in the United States during the period 2006–2010 and to describe the extent to which the observed associations may vary by air temperature. To accomplish these objectives, our study included two major sets of analyses. First, we conducted a main analysis of associations between PM_2.5_ and asthma exacerbations in which PM_2.5_ was evaluated using quartiles, linear splines, and a single continuous measure. Second, we stratified the main analysis into quintiles of air temperature to explore effect modification of the main results by ambient air temperature.

## Methods

### Asthma Call-back Survey

We conducted these analyses using data from the BRFSS adult Asthma Call-back Survey for the years 2006–2010. The BRFSS is a state-level survey of the adult civilian, noninstitutionalized population ≥ 18 years of age that is conducted annually in the United States (CDC 2009). BFRSS survey interviews are conducted throughout the year (i.e., January through December). The Asthma Call-back Survey is a follow-up telephone survey conducted approximately 2 weeks after the BRFSS among respondents who indicated that they have ever had asthma. Respondents reported ever having had asthma by responding “yes” to the following question: “Have you ever been told by a doctor, nurse, or other health professional that you had asthma?” The Asthma Call-back Survey was administered to 10,801 respondents in 2006; 15,245 respondents in 2007; 15,007 in 2008; 15,403 in 2009; and 17,753 in 2010. In the participating areas included in our analysis, the Council of American Survey and Research Organization response rates for the Asthma Call-back Survey ranged from 41% to 71% in 2006, 36% to 72% in 2007, 35% to 68% in 2008, 36% to 66% in 2009, and 31% to 67% in 2010 (Mirabelli et al. 2014; National Asthma Control Program 2011a, 2011b, 2012). The Asthma Call-back Survey is exempt from Institutional Review Board (IRB) review at the CDC; state-specific IRB requirements apply to each of the participating states, the District of Columbia, and Puerto Rico. The protocol for the present analysis was reviewed and determined to be exempt from IRB review at the CDC.

### Study Sample

For this analysis, we present results based on a sample of 50,356 Asthma Call-back Survey respondents. The sample was generated by pooling data collected from Asthma Call back-Surveys conducted in 2006, 2007, 2008, 2009, and 2010. The pooled sample included 74,209 respondents from 42 geographic areas of the United States (40 states, the District of Columbia, and Puerto Rico). We limited our analysis to respondents with active asthma (*n* = 56,509; 76%). We then excluded respondents for whom county- and date-linked air quality data were unavailable (*n* = 3,200, including all respondents from Alaska, Hawaii, and Puerto Rico) and respondents with missing data for the asthma symptom questions and covariates included in our final analysis (*n* = 1,998). Finally, to reduce the influence of the few observations at the lower and upper tails of the distribution of 14-day average PM_2.5_, we excluded from our analyses 418 (1%) observations with PM_2.5_ < 4.00 μg/m^3^ and 537 (1%) observations with PM_2.5_ > 20.00 μg/m^3^. The values of 4.00 μg/m^3^ and 20.00 μg/m^3^ were selected as the minimum and maximum values using the 1st and 99th percentile values of 4.09 μg/m^3^ and 20.12 μg/m^3^, respectively, of the distribution of 14-day average PM_2.5_, rounded to the nearest integer.

### County-level Estimates of Environmental Variables

For this analysis, we linked data from the Asthma Call-back Survey with county-level estimates of environmental variables. In the United States, counties (or equivalent entities such as boroughs, parishes, and independent cities) are the legally defined political and administrative units within each state (U.S. Census Bureau 1994). Among the geographic areas included in our analysis, the number of counties (or equivalent entities, hereafter referred to as “counties”) ranged from 1, in the District of Columbia, to 254, in Texas; in total, we linked environmental data from 2,253 U.S. counties for this analysis. Daily estimates of PM_2.5_ and O_3_ generated using a Bayesian space-time Downscaler fusion model (Berrocal et al. 2012). The Downscaler modeling approach combines output from the Community Multi-scale Air Quality (CMAQ) model (Byun and Schere 2006; Foley et al. 2010) with measurements from the U.S. Environmental Protection Agency’s (EPA’s) Air Quality System (CDC 2013; U.S. EPA 2014) to yield air quality predictions at specific geographic locations. The CMAQ model is a multi-pollutant, multi-scale chemical transport model used to generate air quality predictions at user-defined spatio-temporal scales, taking into account land use, chemical transport, chemistry, emission processes, land use, and weather (Byun and Schere 2006; Foley et al. 2010). Descriptions of the theory, development, and initial evaluation of the downscaler model have been published previously (Berrocal et al. 2010a, 2010b, 2012). Daily predictions of 24-hr average PM_2.5_ concentrations in micrograms/cubic meter and daily maximum 8-hr average O_3_ concentrations in parts per billion were generated at 2010 U.S. census-tract centroid locations (U.S. Census Bureau 2012) using Downscaler software (Heaton et al. 2012). In addition, daily county-level estimates of PM_2.5_ and O_3_ were generated using a population-weighted approach in which census-tract population counts were used to weight daily census tract–level PM_2.5_ and O_3_ predictions (Ivy et al. 2008; Vaidyanathan et al. 2013). County-level estimates of precipitation in millimeters and ambient air temperature in degrees Fahrenheit (°F) were generated using meteorological predictions from the North American Land Data Assimilation System Phase 2 model (Mitchell et al. 2004) at 0.125° spatial resolution (i.e., ~14 × 14 km).

At the time these analyses were conducted, estimates of environmental variables were not available for interview dates in 2011 and, as noted above, all survey respondents for whom environmental data were missing were excluded from analysis. For each of the remaining respondents in our analysis, we assigned 14-day estimates of PM_2.5_, O_3_, precipitation, and temperature using his/her county of residence and the 14-day period leading up to and including the date on which the Asthma Call-back Survey interview was conducted.

For PM_2.5_, we assigned a county-level average of the daily 24-hr average PM_2.5_ concentrations estimated for the 14-day period leading up to and including the day of the interview. Without an *a priori* hypothesis about the best metric of PM_2.5_ to use, we categorized the distribution of 14-day county-level average PM_2.5_ using three metrics: *a*) quartiles (quartile 1, 4.00–7.06 μg/m^3^; quartile 2, 7.07–8.97 μg/m^3^; quartile 3, 8.98–11.36 μg/m^3^; quartile 4, 11.37–19.98 μg/m^3^), *b*) linear spline segments specified with knots between quartiles, and *c*) a single continuous measure of PM_2.5_.

Estimates of O_3_, precipitation, and temperature were each assigned using county-level averages of values estimated for the 14-day period leading up to and including the day of the interview. For O_3_, we assigned a county-level average of the daily 8-hr maximum O_3_ concentrations estimated for the 14-day period leading up to and including the day of the interview. Our final models included O_3_ parameterized using deciles of the distribution, with the 6th decile (range: 38.6–41.4 ppb) as the referent category; the 6th decile was selected as the referent because it included the mean value of the distribution of O_3_ {mean: 38.7 [standard deviation (SD): 9.7]; range: 10.3–83.8 ppb}. Our final models included precipitation in the following categories: 0.0 mm, 0.1–1.1 mm, 1.2–3.0 mm, and ≥ 3.1 mm, with 0.0 mm as the referent category; estimated precipitation > 0.0 mm was categorized as tertiles of the distribution. As with O_3_, our final models included temperature as deciles of the distribution of maximum temperature, with the 5th decile as the referent [range: 58.8–64.6°F (14.9–18.1°C)]; this category was selected as the referent because it included the mean value of the distribution of temperature {mean (SD): 62.9°F (19.3) [17.2°C (10.7)]; range: 1.1–112.4°F (–17.2 to 44.7°C)}.

### Asthma

As in previous analyses (Mirabelli et al. 2014), respondents were categorized as having active asthma if they reported that at least one of the following occurred during the past 12 months: talked to a doctor or other health professional about (his/her) asthma, took asthma medication, or experienced any symptoms of asthma. We used responses to the following questionnaire item as an indicator of the presence of asthma symptoms in the past 2 weeks: “During the past 2 weeks, on how many days were you completely symptom-free—that is, no coughing, wheezing, or other symptoms of asthma?” Respondents who reported being symptom-free on < 14 of the past 14 days were categorized as having asthma symptoms in the past 2 weeks.

### Other Covariates

Demographic covariates used in this analysis include age, educational attainment, race/ethnicity, and sex. Cigarette smoking status was categorized as current smoker, former smoker, and lifetime nonsmoker. The states represented in our final sample were grouped into eight U.S. climate regions classified by the National Climatic Data Center (Karl and Koss 1984; National Climatic Data Center 2014): central (Illinois, Indiana, Missouri, Ohio, West Virginia), east north central (Iowa, Michigan, Wisconsin), northeast (Connecticut, District of Columbia, Maine, Maryland, Massachusetts, New Hampshire, New Jersey, New York, Pennsylvania, Rhode Island, Vermont), northwest (Oregon, Washington), south (Kansas, Louisiana, Mississippi, Oklahoma, Texas), southeast (Alabama, Florida, Georgia, Virginia), southwest (Arizona, Colorado, New Mexico, Utah), west (California, Nevada), and west north central (Montana, Nebraska, North Dakota). The urbanicity of each county in our final sample was categorized as rural, suburban, or urban using 2013 Economic Research Service rural-urban continuum codes published by the U.S. Department of Agriculture (USDA 2013), with counties not adjacent to a metro area categorized as rural, counties adjacent to a metro area categorized as suburban, and counties in metro areas categorized as urban.

### Statistical Analysis

Demographic characteristics and smoking status are presented for the sample of respondents and the weighted population estimate. Weighted population estimates were generated using adjusted sampling weights applied to account for BRFSS and Asthma Call-back Survey nonresponse and unequal sampling probabilities. Annual Asthma Call-back Survey sampling weights were provided with the Asthma Call-back Survey data. Because we pooled data collected from 2006 through 2010 and because the number of geographic areas with Asthma Call-back Survey respondents varied from year to year, we readjusted the sampling weights in each geographic area by dividing the annual Asthma Call-back Survey weights by the number of years for which data were available (Mirabelli et al. 2014). All descriptive analyses were performed using survey procedures (e.g., PROC SURVEYFREQ) for the analysis of complex survey data in SAS v.9.3 (SAS Institute, Inc.).

Using additive binomial models specified with an identity link, we estimated the prevalence of asthma symptoms during the past 14 days for the entire weighted population estimate and across categories of individual- and county-level characteristics, accounting for complex survey sampling. Associations between PM_2.5_ and asthma symptoms during the past 14 days were evaluated using PROC SURVEYREG in SAS v.9.3, with standard errors generated by adapting a two-step approach developed and published by Natarajan and colleagues designed to generate robust variance estimates to fit the binomial error distribution of our data (Natarajan et al. 2008; Slade et al. 2012). Because we conducted our analyses using statistical software designed to analyze complex survey data, all models accounted for the state-based sampling approach and our pooling of 5 years of survey data. Models were adjusted for individual-level covariates (age, educational attainment, race/ethnicity, sex, and smoking status) and county-level covariates (O_3_, precipitation, region, temperature, and urbanicity). After performing these analyses, we conducted two additional analyses in which we replaced the measure of ambient air temperature, initially based on the mean of the distribution of daily maximum temperatures during the 14-day period, with the median of the distribution and with the mean apparent temperature during the 14-day period.

To evaluate the extent to which the observed associations between PM_2.5_ and asthma symptoms in the past 14 days varied by temperature, we stratified adjusted models into quintiles of the distribution of daily maximum ambient air temperature [1.1–44.4°F (–17.2 to 6.8°C), 44.5–58.6°F (6.9–14.8°C), 58.7–70.1°F (14.9–21.1°C), 70.2–80.5°F (21.2–26.9°C), and 80.6–112.4°F (27.0–44.7°C)].

Measures of association are presented as adjusted percent differences (PDs) with 95% confidence intervals (CIs). For analyses in which PM_2.5_ was evaluated using quartiles, PDs are interpreted as arithmetic differences in the prevalence of asthma symptoms in PM_2.5_ quartiles 2, 3, and 4 compared with the prevalence of asthma symptoms in referent quartile 1. For analyses of linear spline segments and analyses in which PM_2.5_ was included as a single continuous measure, PDs are interpreted as the change in prevalence of asthma symptoms per 1 μg/m^3^ increase in PM_2.5_. All analyses were conducted using SAS v.9.3 (SAS Institute, Inc., Cary, North Carolina).

## Results

Characteristics of the 50,356 adults with active asthma and the weighted population estimate are shown in Table 1. Among adults with active asthma, an estimated 57.1% reported asthma symptoms within the past 14 days. Variations in percentages of adults reporting asthma symptoms within the past 14 days were observed across categories of respondent characteristics as well as by U.S. climate region and county-level urbanicity.

**Table 1:** Characteristics of the study sample and the population estimate, with percentages reporting asthma symptoms in the past 14 days.

Characteristic	Survey sample	Weighted population estimate		Asthma symptoms^*a*^
	*n*	*n*^*b*^	Percent (95% CI)	Percent ± SE
Total	50,356	17,963		57.1 ± 0.5
Age, years
18–34	5,430	5,597	31.2 (30.0, 32.4)	51.1 ± 1.3
35–44	6,653	3,318	18.5 (17.7, 19.3)	57.1 ± 1.2
45–54	11,237	3,515	19.6 (18.9, 20.2)	61.0 ± 0.9
55–64	13,075	2,833	15.8 (15.2, 16.3)	61.2 ± 0.8
65–99	13,961	2,701	15.0 (14.5, 15.5)	60.4 ± 0.8
Educational attainment
Less than high school	4,795	1,817	10.1 (9.5, 10.7)	63.2 ± 1.6
Graduated high school	13,372	4,628	25.8 (24.8, 26.8)	60.2 ± 1.2
College 1–3 years/technical school	15,062	5,120	28.5 (27.6, 29.4)	59.5 ± 1.0
College ≥ 4 years	17,127	6,398	35.6 (34.6, 36.6)	51.4 ± 0.8
Race/ethnicity
White, non-Hispanic	42,143	13,415	74.7 (73.6, 75.7)	58.3 ± 0.6
Black, non-Hispanic	3,105	1,656	9.2 (8.6, 9.9)	55.4 ± 1.9
Other, non-Hispanic	2,812	1,161	6.5 (5.9, 7.1)	61.8 ± 2.5
Hispanic	2,296	1,731	9.6 (8.8, 10.5)	46.8 ± 2.4
Sex
Female	36,995	11,244	62.6 (61.5, 63.7)	58.0 ± 0.6
Male	13,361	6,719	37.4 (36.3, 38.5)	55.8 ± 1.0
Smoking status
Current smoker	9,261	3,473	19.3 (18.5, 20.1)	69.8 ± 1.1
Former smoker	17,085	4,877	27.1 (26.3, 28.0)	59.6 ± 0.9
Lifetime nonsmoker	24,010	9,613	53.5 (52.5, 54.5)	51.3 ± 0.8
U.S. climate region
Central	5,901	2,687	15.0 (14.4, 15.5)	60.8 ± 1.2
East north central	4,575	1,401	7.8 (7.5, 8.1)	61.2 ± 1.2
Northeast	13,481	4,582	25.5 (24.8, 26.3)	56.2 ± 1.1
Northwest	5,943	741	4.1 (4.0, 4.3)	59.6 ± 1.2
South	6,478	2,226	12.4 (11.8, 13.0)	62.1 ± 1.6
Southeast	3,442	2,423	13.5 (12.9, 14.1)	59.0 ± 1.6
Southwest	3,778	1,029	5.7 (5.4, 6.0)	60.7 ± 1.8
West	2,587	2,676	14.9 (14.2, 15.6)	45.0 ± 1.7
West north central	4,171	198	1.1 (1.0, 1.2)	57.9 ± 1.5
Urbanicity
Rural	7,329	1,007	5.6 (5.3, 5.9)	63.1 ± 1.5
Suburban	9,238	2,122	11.8 (11.3, 12.4)	65.1 ± 1.2
Urban	33,789	14,834	82.6 (82.0, 83.2)	55.6 ± 0.6
Notes: CI, confidence interval; SE, standard error of the mean. ^***a***^In the past 14 days. ^***b***^In thousands.				

**Figure 1:**
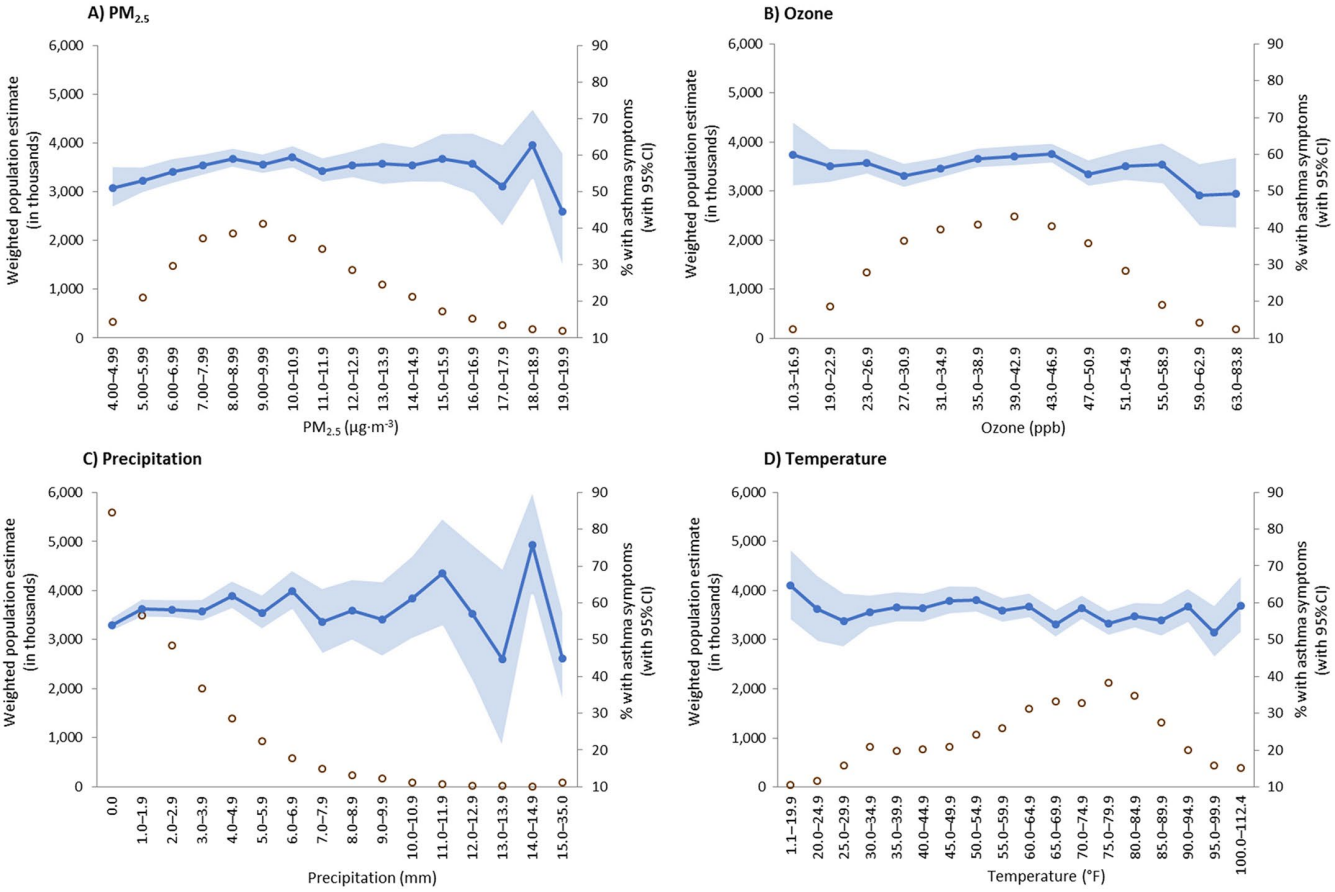
Weighted estimates of the population with active asthma (open circles) and estimated percentages of adults with asthma symptoms in the past 14 days (filled circles) across categories of particulate matter ≤ 2.5 μm in diameter (PM_2.5_) (*A*), ozone (*B*), precipitation (*C*), and temperature (*D*). Formula for conversion from degrees Fahrenheit (T_F_) to degrees Celsius (T_C_): T_C_ = (T_F_ – 32) × (5/9).

Summary statistics describing the distributions of PM_2.5_, O_3_, temperature, and precipitation as well as Pearson’s correlation coefficients indicating correlations between the four measures are shown in Table 2. Despite the small magnitudes of the correlations, all pairwise correlation coefficients were statistically significant at α < 0.05. The distributions of PM_2.5_, O_3_, precipitation, and temperature for the weighted population estimate of nearly 18.0 million adults with active asthma and the estimated percentages of adults with active asthma reporting asthma symptoms in the past 14 days across categories of each environmental measure are shown in Figure 1. Across quartiles of PM_2.5_, the percentages of adults reporting asthma symptoms in the past 14 days were 54.2% ± 1.2 in quartile 1, 58.4% ± 1.0 in quartile 2, 58.3% ± 1.0 in quartile 3, and 56.7% ± 1.1 in quartile 4.

**Table 2:** Summary statistics and correlation coefficients describing the distributions of PM_2.5_, O_3_, precipitation, and temperature.

Exposure	*n*	Average daily mean ± SD^*a*^	Percentiles^*a*^			Pearson’s correlation coefficients^*a*^			
			25th	50th	75th	PM_2.5_	O_3_	Precipitation	Temperature
PM_2.5_ (μg/m^3^)	50,356	9.4 ± 3.1	7.1	9.0	11.4	1	0.03	–0.02	–0.02
O_3_ (ppb)	50,356	38.7 ± 9.7	31.2	38.6	45.7		1	–0.11	0.57
Precipitation (mm)	50,356	2.8 ± 3.3	0.8	2.0	3.7			1	–0.04
Temperature [°F (°C)]	50,356	62.9 (17.2) ± 19.3 (10.7)	48.5 (9.1)	64.6 (18.1)	77.9 (25.5)				1
Notes: O_3_, ozone; PM_2.5_, particulate matter ≤ 2.5 μm in diameter; SD, standard deviation. ^***a***^Based on unweighted survey data.									

Table 3 shows estimates of the difference in the percentage of adults with active asthma who experienced symptoms in the past 14 days; the values were generated using unadjusted models and models adjusted for individual- and county-level covariates. Broadly speaking, estimates generated using models adjusted for individual-level covariates were similar to those generated using unadjusted models, and estimates generated using models adjusted for county-level covariates were similar to those generated using fully adjusted models. In fully adjusted models, when PM_2.5_ exposure was parameterized using indicators of PM_2.5_ quartile, the estimated prevalences of asthma symptoms in the past 14 days were typically 4–5% higher in quartiles 2, 3, and 4 than the prevalence in quartile 1. In models adjusted for individual- and county-level covariates in which PM_2.5_ was parameterized as four linear spline segments, percent differences in 14-day symptom prevalence per 1 μg/m^3^ increase in PM_2.5_ were 3.4% (95% CI: 1.1, 5.7) in segment 1, 0.9 (95% CI: –1.3, 3.1) in segment 2, –0.1 (95% CI: –1.6, 1.5) in segment 3, and 0.3 (95% CI: –0.6, 1.1) in segment 4. These PDs indicate an estimated increase of 3.4% of adults with active asthma reporting symptoms in the past 14 days with each 1 μg/m^3^ unit increase in PM_2.5_ between 4.00 and 7.06 μg/m^3^ and minimal change per unit increase from 7.07 to 19.98 μg/m^3^. In contrast, when we considered adjusted associations with each 1 μg/m^3^ unit increase in PM_2.5_ across the entire distribution of PM_2.5_, our models generated a per-unit increase in symptom prevalence of 0.5% (95% CI: 0.1, 0.9).

**Table 3:** Associations between PM_2.5_ and the prevalence of asthma symptoms in the past 14 days among adults with active asthma.

Metrics of PM_2.5_	Survey sample	Weighted population estimate	Unadjusted model	Partially adjusted models		Fully adjusted, final model
	*n*	*n*^*a*^	PD (95% CI)	PD (95% CI)^*b*^	PD (95% CI)^*c*^	PD (95% CI)^*b*^^,^^*c*^
Quartiles
4.00–7.06 μg/m^3^	12,640	2,747	0.0 (Referent)	0.0 (Referent)	0.0 (Referent)	0.0 (Referent)
7.07–8.97 μg/m^3^	12,586	4,054	4.2 (1.1, 7.3)	4.2 (1.2, 7.1)	4.5 (1.4, 7.6)	4.4 (1.4, 7.4)
8.98–11.36 μg/m^3^	12,595	5,160	4.1 (1.0, 7.1)	4.3 (1.4, 7.2)	4.6 (1.4, 7.8)	4.7 (1.6, 7.8)
11.37–19.98 μg/m^3^	12,535	6,002	2.5 (–0.6, 5.6)	3.0 (0.1, 5.9)	4.8 (1.3, 8.3)	4.9 (1.5, 8.2)
Linear spline segments
Per μg/m^3^ 4.00–7.06	12,640	2,747	2.9 (0.6, 5.2)	3.1 (0.8, 5.3)	3.4 (1.1, 5.7)	3.4 (1.1, 5.7)
Per μg/m^3^ 7.07–8.97	12,586	4,054	0.9 (–1.4, 3.2)	0.8 (–1.3, 3.0)	0.9 (–1.4, 3.1)	0.9 (–1.3, 3.0)
Per μg/m^3^ 8.98–11.36	12,595	5,160	–0.6 (–2.2, 1.1)	–0.5 (–2.0, 1.1)	0.0 (–1.7, 1.6)	–0.1 (–1.6, 1.5)
Per μg/m^3^ 11.37–19.98	12,535	6,002	–0.2 (–1.1, 0.8)	–0.1 (–1.0, 0.8)	0.2 (–0.7, 1.2)	0.3 (–0.6, 1.1)
Continuous measure
Per μg/m^3^ 4.00–19.98	50,356	17,963	0.2 (–0.2, 0.6)	0.2 (–0.1, 0.5)	0.5 (0.1, 0.9)	0.5 (0.1, 0.9)
Notes: CI, confidence interval; O_3_, ozone; PD, percent difference; PM_2.5_, particulate matter ≤ 2.5 μm in diameter. ^***a***^In thousands. ^***b***^Adjusted for individual-level covariates: age, educational attainment, race, sex, and smoking status. ^***c***^Adjusted for county-level covariates: O_3_, precipitation, region, temperature, and urbanicity.						

These results were robust to changes in the measure of ambient air temperature. Our main analyses included ambient air temperature parameterized using the mean of the distribution of 14-day average daily maximum temperatures. Replacing this measure with variables parameterized using the median of the distribution or the mean apparent temperature generated estimates of effect that were nearly identical in magnitude and precision (data not shown).

**Figure 2:**
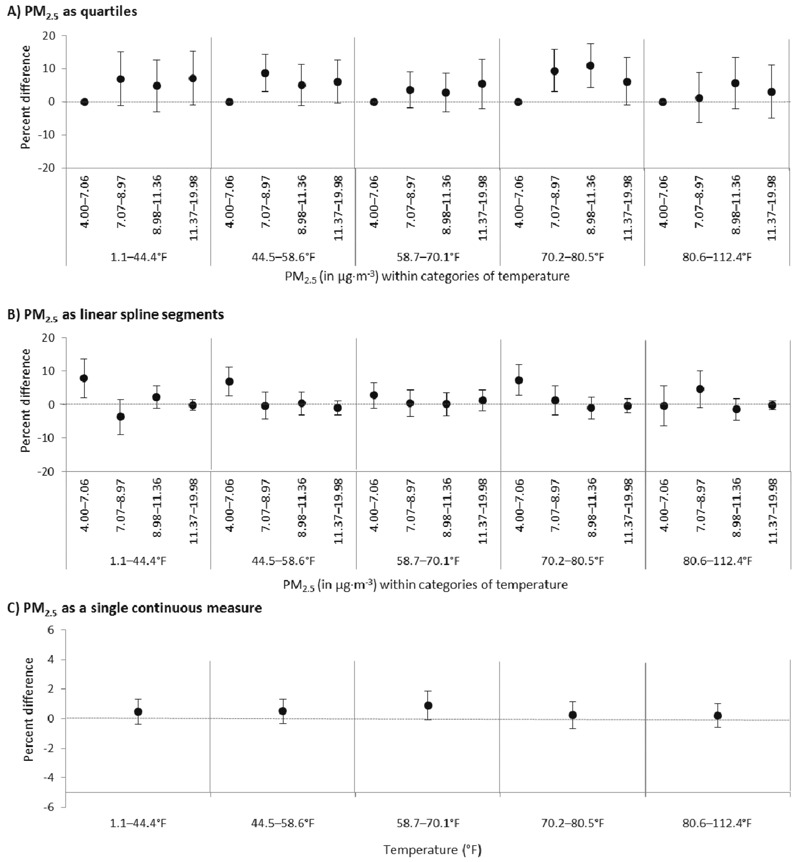
Associations between particulate matter ≤ 2.5 μm in diameter (PM_2.5_) and the prevalence of asthma symptoms in the past 14 days among adults with active asthma across quintiles of air temperature. Results are shown for temperature category-stratified models in which PM_2.5_ is parameterized as quartiles (*A*), four linear spline segments (*B*), and a single continuous measure (*C*). All models are adjusted for individual- and county-level covariates. Formula for conversion from degrees Fahrenheit (T_F_) to degrees Celsius (T_C_): T_C_ = (T_F_ – 32) × (5/9).

Variations in the estimates across categories of air temperature are shown in Figure 2. When PM_2.5_ exposure was modeled using quartiles, estimated differences in the prevalence of asthma symptoms in the past 14 days were similar across all categories of temperature and highest in the 70.2–80.5°F (21.2–26.9°C) range [quartile 2: 9.4% (95% CI: 3.1, 15.7), quartile 3: 11.0% (95% CI: 4.4, 17.6), and quartile 4: 6.1% (95% CI: –1.1, 13.3)] (see Table S1). When PM_2.5_ exposure was parameterized as linear spline segments, the pattern observed in our main analysis was also observed across quintiles of temperature. Spline segment 1 was associated with positive point estimates of the PD per microgram/cubic meter of PM_2.5_ across categories of temperature ranging from 1.1°F to 80.5°F (–17.2 to 26.9°C) [1.1–44.4°F (–17.2 to 6.8°C): 7.9%; 44.5–58.6°F (6.9–14.7°C): 6.9%; 58.7–70.1°F (14.8–21.1°C): 2.9%; 70.2–80.5°F (21.2–26.9°C): 7.3%]. Per-microgram/cubic meter changes in symptom prevalence in spline segments 2 through 4 were consistent with the null value of 0.0%. Minimal variation was observed across categories of temperature when PM_2.5_ was evaluated as a single continuous measure.

## Discussion

Our findings suggest that among adults with asthma, the prevalence of self-reported asthma symptoms during the past 14 days was 4–5% higher among respondents with a 14-day average concentration of PM_2.5_ > 7.07 μg/m^3^ than the prevalence among respondents with 14-day averages in the range of 4.00–7.06 μg/m^3^_._ In this lowest category of PM_2.5_, each 1 μg/m^3^ increase in PM_2.5_ was associated with a 3% increase in the prevalence of asthma symptoms. Taken together, our results raise the possibility that among individuals with asthma, exacerbations may begin to increase at relatively low levels of ambient PM_2.5_. Stratification of these results across categories of temperature suggests that these findings may be driven largely by effects observed at temperatures < 80.6°F (27.0°C).

These results expand on previous research that evaluated relationships between PM_2.5_ and asthma symptoms across quartiles of the distribution of PM_2.5_ (Mirabelli et al. 2015) by exploring additional metrics of PM_2.5_ and refining the statistical models used to estimate the exposure–outcome relationships. Previously, we reported on quartiles of the PM_2.5_ distribution and found higher percentages of adults with asthma symptoms in the past 14 days in quartiles 2, 3, and 4 compared with quartile 1 (Mirabelli et al. 2015). In considering relationships between PM_2.5_ and asthma symptoms using four linear spline segments, the present analysis supports and expands our earlier findings by suggesting that each unit increase in PM_2.5_ may be associated with a measurable increase in the prevalence of asthma symptoms in the past 14 days, even at levels as low as 4.00–7.06 μg/m^3^. Indeed, using results from our fully adjusted linear spline segment model, PD estimates generated at the median of each spline segment support the results of our analysis using quartiles. For example, where models using quartiles of PM_2.5_ generated PDs of 4.4%, 4.7%, and 4.9% in quartiles 2, 3, and 4, respectively, models using linear spline segments generated PDs of 4.5%, 5.2%, and 5.7% (data not shown). Both models suggest that among individuals with asthma, exacerbations may begin to increase at levels of PM_2.5_ < 7.07 μg/m^3^. The strength of this association at levels between 4.00 and 7.06 μg/m^3^ would have been missed had we only evaluated the relationship between PM_2.5_ and asthma symptoms using a single continuous measure of PM_2.5_, which generated a 0.5% increase in the prevalence of asthma symptoms with each 1 μg/m^3^ increase in PM_2.5_.

At the present time, there are few population-based studies of PM_2.5_ and asthma morbidity outcomes with which to contrast our results. Following an evaluation of associations between daily minimum temperature and respiratory hospitalizations and emergency department visits, investigators reported that ambient concentrations of PM ≤ 10 μm in aerodynamic diameter (PM_10_) modified the association between temperature and respiratory hospitalizations but not emergency department visits, and that the observed associations with increasing temperatures were more pronounced when concentrations of PM_10_ also increased (Ren et al. 2006). Combined with convincing evidence of the effects of ambient PM exposures on the respiratory health of adults [Health Effects Institute (HEI) Panel on the Health Effects of Traffic-Related Air Pollution 2010], our findings that the magnitudes of the associations between PM_2.5_ and asthma symptoms are not constant across the range of 4.00–19.98 μg/m^3^ of PM_2.5_ and that the associations vary across categories of ambient air temperature support and extend previous evidence that ambient particulate matter pollution and temperature may act jointly to affect respiratory health in manners that may be evident before death (Ren et al. 2006).

Several aspects of our study merit careful consideration when interpreting our findings. First, a comparison of the results shown in Table 1 suggests that the relationship between PM_2.5_ and the prevalence of asthma symptoms in the past 14 days may be confounded by county-level factors, including O_3_, precipitation, and temperature. Despite including these and other county-level covariates in our analysis, our results may be affected by residual confounding, including within categories of ambient air temperature or by geographic differences within climate regions. We were unable to stratify our results into smaller categories of air temperature. Earlier research designed to estimate the effects of ambient air pollutant exposures on health initially suggested that statistical models that include one or two weather variables, such as temperature and humidity, may not be adequate to fully control for the effects of weather (Pope and Kalkstein 1996). From subsequent research designed to evaluate the extent to which statistical models of the relationship between particulate pollution and mortality that simultaneously account for the effects of weather or temperature may yield biased results if incorrect metrics of weather or temperature are used, investigators reported finding little evidence that the choice of metrics influenced findings (Samet et al. 1998). Results generated from our main models, which included temperature and O_3_ using indicators for deciles of the distributions and precipitation using indicators for tertiles of the distribution, may not have adequately accounted for the complex relationships between air quality, weather, and health, but they were not notably changed when we considered alternative parameterizations of O_3_, temperature, and precipitation (not shown). Improvements in our understanding of the complex relationships between air quality, weather, and health would improve our ability to incorporate these relationships, including nonlinear relationships and relationships at temperature extremes (Pope and Kalkstein 1996), into our analyses of the relationship between air quality and asthma exacerbations.

Second, modeled exposures provide estimates of PM_2.5_, O_3_, precipitation, and temperature for geographic areas in which respondents live. Assigning county-level exposure measures may have resulted in exposure misclassification if respondents’ true exposures were markedly different from those assigned to the counties in which they lived. The modeling approach applied in our study advantageously provided estimates for geographic areas without adequate measurements of air pollutants or meteorological parameters. However, uncertainties associated with estimates of exposures derived using modeled data could result in exposure misclassification, which is not incorporated into our statistical models, and we were unable to take into account a wide range of other air pollutants or PM of other sizes (e.g., coarse or ultrafine). When concentrations of multiple air pollutants are highly correlated, results generated from models that do not include each of the multiple pollutants may be confounded by the effects of other pollutants not considered, and we are unable to evaluate the extent to which our findings may represent relationships between other air pollutants and exacerbations of asthma. Nonetheless, the integration of data from a large, population-based survey of adults with asthma with environmental data estimated at the county level enabled us to evaluate the relationship between county-level PM_2.5_ and asthma symptoms in a large population of U.S. adults.

Our analyses did not account for indoor exposures, filtration of indoor air, air exchange rates, occupational exposures, pollen concentrations, or other factors affecting personal exposure. We did not have information about the extent to which respondents may have been included in two or more annual sample populations. Our analyses did not take into account the timing, including day of the week, month, or season, of the survey interview. In these data, correlations between interview month, U.S. climate region, and temperature prevented our statistical models from converging when all three factors were included. Similarly, imprecise estimates within each state prevented us from stratifying our analysis by state. When using these data, we were unable to evaluate temporal relationships between exposures and asthma symptoms within the 14-day period; therefore, we cannot draw conclusions about the extent to which symptoms may have preceded or followed peak exposures. Furthermore, our analyses did not incorporate information about the changes in exposure to outdoor air or changes in behaviors that may occur at temperature extremes. If any behavioral changes, such as closing windows, using air conditioning, and staying indoors during hot weather, reduced exposures to ambient air pollutants, then our observation of attenuated associations at temperatures > 80.6°F (27.0°C) is unsurprising. In addition to the direct effects of exposure to ambient particulate matter pollution, susceptibility to the effects of exposure may be linked to other factors associated with asthma, such as diet and obesity, indoor air quality, medication use, socioeconomic status, and stress (Guarnieri and Balmes 2014). Some populations, such as those living in neighborhoods located near highways or affected by industrial air emissions, may experience exposures higher than county averages and be more vulnerable to the effects of poor air quality. Additional information about other factors (e.g., pollen and other allergens) associated with exacerbation of asthma and about the frequency and severity of the asthma exacerbations would improve our ability to draw conclusions about the role of exposures to PM_2.5_ in ambient air.

## Conclusion

Despite the limitations described above, our findings extend the present understanding of the health effects of PM_2.5_ by considering self-reported asthma exacerbations rather than more severe outcomes such as hospital encounters or mortality. Health effects associated with PM_2.5_ exposures are wide-ranging and may have measurable impacts on outcomes not considered in this analysis, including indicators of asthma severity, symptom frequency, medication use, functional consequences of asthma, and other cardiovascular and respiratory conditions. Our results suggest that the relationship between PM_2.5_ and asthma may not be constant across the entire range of PM_2.5_ concentrations, and as a consequence, when the exposure of interest is PM_2.5_ in ambient air, changes in population-level metrics of asthma exacerbations may occur most noticeably in the range of 4.00–7.06 μg/m^3^_._ These findings provide novel information about the importance of county-level air quality for the nearly 18 million adults with asthma represented by the sampled survey population of Asthma Call-back Survey respondents.

## Supplemental Material

(54 KB) PDF

## References

[r1] Anderson SD, Daviskas E (2000). The mechanism of exercise-induced asthma is.... J Allergy Clin Immunol.

[r2] Andrade H, Alcoforado MJ, Oliveira S (2011). Perception of temperature and wind by users of public outdoor spaces: relationships with weather parameters and personal characteristics.. Int J Biometeorol.

[r3] Balmes JR, Cisternas M, Quinlan PJ, Trupin L, Lurmann FW, Katz PP (2014). Annual average ambient particulate matter exposure estimates, measured home particulate matter, and hair nicotine are associated with respiratory outcomes in adults with asthma.. Environ Res.

[r4] Baur D, Saisana M, Schulze N (2004). Modelling the effects of meteorological variables on ozone concentration—a quantile regression approach.. Atmos Environ.

[r5] Beard JD Beck C Graham R Packham SC Traphagan M Giles RT 2012 Winter temperature inversions and emergency department visits for asthma in Salt Lake County, Utah, 2003–2008. Environ Health Perspect 120 1385 1390, doi:10.1289/ehp.1104349 22784691 PMC3491922

[r6] Bell ML, Ebisu K, Peng RD, Walker J, Samet JM, Zeger SL (2008). Seasonal and regional short-term effects of fine particles on hospital admissions in 202 US counties, 1999–2005.. Am J Epidemiol.

[r7] Belleudi V, Faustini A, Stafoggia M, Cattani G, Marconi A, Perucci CA (2010). Impact of fine and ultrafine particles on emergency hospital admissions for cardiac and respiratory diseases.. Epidemiology.

[r8] Bernstein JA, Alexis N, Barnes C, Bernstein IL, Nel A, Peden D (2004). Health effects of air pollution.. J Allergy Clin Immunol.

[r9] Berrocal VJ, Gelfand AE, Holland DM (2010a). A bivariate space-time downscaler under space and time misalignment.. Ann Appl Stat.

[r10] Berrocal VJ, Gelfand AE, Holland DM (2010b). A spatio-temporal downscaler for output from numerical models.. J Agric Biol Environ Stat.

[r11] Berrocal VJ, Gelfand AE, Holland DM (2012). Space-time data fusion under error in computer model output: an application to modeling air quality.. Biometrics.

[r12] Breitner S, Wolf K, Devlin RB, Diaz-Sanchez D, Peters A, Schneider A (2014). Short-term effects of air temperature on mortality and effect modification by air pollution in three cities of Bavaria, Germany: a time-series analysis.. Sci Total Environ.

[r13] Byun D, Schere KL (2006). Review of the governing equations, computational algorithms, and other components of the Models-3 Community Multiscale Air Quality (CMAQ) modeling system.. Appl Mech Rev.

[r14] Camalier L, Cox W, Dolwick P (2007). The effects of meteorology on ozone in urban areas and their use in assessing ozone trends.. Atmos Environ.

[r15] CDC (Centers for Disease Control and Prevention) (2009). Behavioral Risk Factor Surveillance System (BRFSS).. http://www.cdc.gov/asthma/survey/brfss.html.

[r16] CDC (2013). Outdoor Air: Monitor + Modeled Air Data.. http://ephtracking.cdc.gov/showAirMonModData.action.

[r17] Dominici F, Peng RD, Bell ML, Pham L, McDermott A, Zeger SL (2006). Fine particulate air pollution and hospital admission for cardiovascular and respiratory diseases.. JAMA.

[r18] Dominici F, Samet JM, Zeger SL (2000). Combining evidence on air pollution and daily mortality from the 20 largest US cities: a hierarchical modeling strategy.. J R Statist Soc A.

[r19] Fitzgerald EF, Pantea C, Lin S (2014). Cold spells and the risk of hospitalization for asthma: New York, USA 1991–2006.. Lung.

[r20] Foley KM, Roselle SJ, Appel KW, Bhave PV, Pleim JE, Otte TL (2010). Incremental testing of the Community Multiscale Air Quality (CMAQ) modeling system version 4.7.. Geosci Model Dev.

[r21] Guarnieri M, Balmes JR (2014). Outdoor air pollution and asthma.. Lancet.

[r22] He H, Hembeck L, Hosley KM, Canty TP, Salawitch RJ, Dickerson RR (2013). High ozone concentrations on hot days: the role of electric power demand and NO_x_ emissions.. Geophys Res Lett.

[r23] Heaton M, Holland DM, Leininger T (2012). *User’s Manual for Downscaler Fusion Software*. TIP#12-017. EPA/600/C-12/002..

[r24] HEI Panel on the Health Effects of Traffic-Related Air Pollution (2010). *Traffic-Related Air Pollution: a Critical Review of the Literature on Emissions, Exposure, and Health Effects*.. HEI Special Report 17. Health Effects Institute, Boston, MA.

[r25] Hernandez ML, Lay JC, Harris B, Esther CR Jr, Brickey WJ, Bromberg PA, et al (2010). Atopic asthmatic subjects but not atopic subjects without asthma have enhanced inflammatory response to ozone.. J Allergy Clin Immunol.

[r26] Ivy D, Mulholland JA, Russell AG (2008). Development of ambient air quality population-weighted metrics for use in time-series health studies.. J Air Waste Manag Assoc.

[r27] Karl TR, Koss WJ (1984). *Regional and National Monthly, Seasonal, and Annual Temperature Weighted by Area, 1895–1983. Historical Climatology Series 4-3*..

[r28] Khatri SB, Holguin FC, Ryan PB, Mannino D, Erzurum SC, Teague WG (2009). Association of ambient ozone exposure with airway inflammation and allergy in adults with asthma.. J Asthma.

[r29] Koren HS (1995). Associations between criteria air pollutants and asthma.. Environ Health Perspect.

[r30] Li G, Zhou M, Cai Y, Zhang Y, Pan X (2011). Does temperature enhance acute mortality effects of ambient particle pollution in Tianjin City, China.. Sci Total Environ.

[r31] Li N, Hao M, Phalen RF, Hinds WC, Nel AE (2003). Particulate air pollutants and asthma. A paradigm for the role of oxidative stress in PM-induced adverse health effects.. Clin Immunol.

[r32] Lim SS, Vos T, Flaxman AD, Danaei G, Shibuya K, Adair-Rohani H (2012). A comparative risk assessment of burden of disease and injury attributable to 67 risk factors and risk factor clusters in 21 regions, 1990–2010: a systematic analysis for the Global Burden of Disease Study 2010.. Lancet.

[r33] Michelozzi P, Accetta G, De Sario M, D’Ippoliti D, Marino C, Baccini M (2009). High temperature and hospitalizations for cardiovascular and respiratory causes in 12 European cities.. Am J Respir Crit Care Med.

[r34] Mirabelli MC, Beavers SF, Chatterjee AB (2014). Active asthma and the prevalence of physician-diagnosed COPD.. Lung.

[r35] Mirabelli MC, Vaidyanathan A, Qin X, Garbe PL (2015). County-level PM_2.5_ and asthma symptoms in the past 14 days among U.S. adults with active asthma [Abstract].. Am J Respir Crit Care Med.

[r36] Mitchell KE Lohmann D Houser PR Wood EF Schaake JC Robock A 2004 The multi-institution North American Land Data Assimilation System (NLDAS): utilizing multiple GCIP products and partners in a continental distributed hydrological modeling system. J Geophys Res 109 D07S90, doi:10.1029/2003JD003823

[r37] Natarajan S, Lipsitz SR, Fitzmaurice G, Moore CG, Gonin R (2008). Variance estimation in complex survey sampling for generalized linear models.. J R Stat Soc Ser C Appl Stat.

[r38] National Asthma Control Program (2011a). 2006–2008 Behavioral Risk Factor Surveillance System Asthma Call-Back Survey Summary Data Quality Report.. http://www.cdc.gov/brfss/acbs/2008/pdf/sdqreportacbs_06-08.pdf.

[r39] National Asthma Control Program (2011b). 2009 Behavioral Risk Factor Surveillance System Asthma Call-Back Survey Summary Data Quality Report.. http://www.cdc.gov/brfss/acbs/2009/documentation/SDQReportACBS_09.rtf.

[r40] National Asthma Control Program (2012). 2010 Behavioral Risk Factor Surveillance System Asthma Call-back Survey Summary Quality Data Report.. http://www.cdc.gov/brfss/acbs/2010/pdf/sdqreportacbs_10.pdf.

[r41] National Climatic Data Center (2014). National Oceanic and Atmospheric Administration. U.S. Climate Regions.. http://www.ncdc.noaa.gov/monitoring-references/maps/us-climate-regions.php.

[r42] Nawrot TS, Torfs R, Fierens F, De Henauw S, Hoet PH, Van Kersschaever G (2007). Stronger associations between daily mortality and fine particulate air pollution in summer than in winter: evidence from a heavily polluted region in western Europe.. J Epidemiol Community Health.

[r43] Nel AE, Diaz-Sanchez D, Li N (2001). The role of particulate pollutants in pulmonary inflammation and asthma: evidence for the involvement of organic chemicals and oxidative stress.. Curr Opin Pulm Med.

[r44] Park AK, Hong YC, Kim H (2011). Effect of changes in season and temperature on mortality associated with air pollution in Seoul, Korea.. J Epidemiol Community Health.

[r45] Pope CA, Kalkstein LS (1996). Synoptic weather modeling and estimates of the exposure-response relationship between daily mortality and particulate air pollution.. Environ Health Perspect.

[r46] Ren C Williams GM Tong S 2006 Does particulate matter modify the association between temperature and cardiorespiratory diseases? Environ Health Perspect 114 1690 1696, doi:10.1289/ehp.9266 17107854 PMC1665419

[r47] Samet JM, Dominici F, Curriero FC, Coursac I, Zeger SL (2000). Fine particulate air pollution and mortality in 20 U.S. cities, 1987–1994.. N Engl J Med.

[r48] Samet J, Zeger S, Kelsall J, Xu J, Kalkstein L (1998). Does weather confound or modify the association of particulate air pollution with mortality? An analysis of the Philadelphia data, 1973–1980.. Environ Res.

[r49] Slade GD, Sanders AE, By K (2012). Role of allostatic load in sociodemographic patterns of pain prevalence in the U.S. population.. J Pain.

[r50] U.S. Census Bureau (1994). States, counties, and statistically equivalent entities. In: *Geographic Areas Reference Manual*.. http://www2.census.gov/geo/pdfs/reference/GARM/Ch4GARM.pdf.

[r51] U.S. Census Bureau (2012). TIGER/Line^®^ Shapefiles and TIGER/Line^®^ Files.. https://www.census.gov/geo/maps-data/data/tiger-line.html.

[r52] U.S. EPA (U.S. Environmental Protection Agency) (2014). Air Quality System (AQS).. https://www.epa.gov/aqs.

[r53] USDA (U.S. Department of Agriculture) (2013). Rural-Urban Continuum Codes: Documentation.. http://www.ers.usda.gov/data-products/rural-urban-continuum-codes/documentation.aspx.

[r54] Vahlsing C, Smith KR (2012). Global review of national ambient air quality standards for PM_10_ and SO_2_ (24 h).. Air Qual Atmos Health.

[r55] Vaidyanathan A Dimmick WF Kegler SR Qualters JR 2013 Statistical air quality predictions for public health surveillance: evaluation and generation of county level metrics of PM_2.5_ for the Environmental Public Health Tracking Network. Int J Health Geogr 12 12, doi:10.1186/1476-072X-12-12 23497176 PMC3601977

[r56] Young MT, Sandler DP, DeRoo LA, Vedal S, Kaufman JD, London SJ (2014). Ambient air pollution exposure and incident adult asthma in a nationwide cohort of U.S. women.. Am J Respir Crit Care Med.

[r57] Zanobetti A Schwartz J 2009 The effect of fine and coarse particulate air pollution on mortality: a national analysis. Environ Health Perspect 117 898 903, doi:10.1289/ehp.0800108 19590680 PMC2702403

