# Supplementary material for: Outdoor PM2.5, Ambient Air Temperature, and Asthma Symptoms in the Past 14 Days among Adults with Active Asthma
Source: Environ Health Perspect. 2016 Jul 6;124(12):1882–90. doi: 10.1289/EHP92 (PMC5132644; doi:10.1289/EHP92)
Supplement: (54 KB) PDF [file EHP92.s001.acco.pdf]

Environ Health Perspect
DOI: 10.1289/EHP92

Note to readers with disabilities: *EHP* strives to ensure that all journal content is accessible to all readers. However, some figures and Supplemental Material published in *EHP* articles may not conform to 508 standards due to the complexity of the information being presented. If you need assistance accessing journal content, please contact ehp508@niehs.nih.gov. Our staff will work with you to assess and meet your accessibility needs within 3 working days.

Supplemental Material

Outdoor $PM_{2.5}$, Ambient Air Temperature, and Asthma Symptoms in the Past 14 Days among Adults with Active Asthma

Maria C. Mirabelli, Ambarish Vaidyanathan, W. Dana Flanders, Xiaoting Qin, and Paul Garbe

Table of Contents

Table S1. Associations between $PM_{2.5}$ and the prevalence of asthma symptoms in the past 14 days among adults with active asthma across quintiles of air temperature

Table S1. Associations between $PM_{2.5}$ and the prevalence of asthma symptoms in the past 14 days among adults with active asthma across quintiles of air temperature.

	Air Temperature (°F)				
	1.1–44.4	44.5–58.6	58.7–70.1	70.2–80.5	80.6–112.4
Metrics of $PM_{2.5}$	PD (95% CI)[a]	PD (95% CI)[a]	PD (95% CI)[a]	PD (95% CI)[a]	PD (95% CI)[a]
**Quartiles**					
4.00–7.06 $\mu g \cdot m^{-3}$	0.0 (referent)	0.0 (referent)	0.0 (referent)	0.0 (referent)	0.0 (referent)
7.07–8.97 $\mu g \cdot m^{-3}$	7.0 (-1.2, 15.1)	8.7 (3.0, 14.3)	3.6 (-1.8, 8.9)	9.4 (3.1, 15.7)	1.2 (-6.3, 8.7)
8.98–11.36 $\mu g \cdot m^{-3}$	4.8 (-3.0, 12.6)	5.0 (-1.2, 11.3)	2.8 (-3.0, 8.7)	11.0 (4.4, 17.6)	5.7 (-2.0, 13.3)
11.37–19.98 $\mu g \cdot m^{-3}$	7.1 (-1.1, 15.3)	6.1 (-0.5, 12.8)	5.5 (-2.0, 13)	6.1 (-1.1, 13.3)	3.1 (-4.9, 11.0)
**Linear spline segments**					
per $\mu g \cdot m^{-3}$ 4.00–7.06	7.9 (1.9, 13.8)	6.9 (2.6, 11.2)	2.9 (-1.0, 6.7)	7.3 (2.7, 11.9)	-0.3 (-6.3, 5.6)
per $\mu g \cdot m^{-3}$ 7.07–8.97	-3.7 (-9.0, 1.6)	-0.3 (-4.4, 3.7)	0.5 (-3.4, 4.3)	1.3 (-3.1, 5.7)	4.6 (-0.9, 10.1)
per $\mu g \cdot m^{-3}$ 8.98–11.36	2.3 (-1.1, 5.7)	0.4 (-3.1, 3.9)	0.2 (-3.2, 3.6)	-1.0 (-4.3, 2.3)	-1.4 (-4.6, 1.9)
per $\mu g \cdot m^{-3}$ 11.37–19.98	-0.1 (-1.6, 1.5)	-0.9 (-3.1, 1.3)	1.3 (-1.9, 4.5)	-0.3 (-2.4, 1.8)	-0.2 (-1.5, 1.2)
**Continuous measure**					
per $\mu g \cdot m^{-3}$ 4.00–19.98	0.5 (-0.4, 1.3)	0.5 (-0.3, 1.3)	0.9 (-0.1, 1.9)	0.2 (-0.7, 1.1)	0.2 (-0.6, 1.0)

CI, confidence interval; PD, percent difference

[a] Adjusted for individual-level covariates (age, educational attainment, race, sex, and smoking status) and county-level covariates (O_3, precipitation, region, temperature, and urbanicity)
